# A Tissue-Compliant Shape-Memory Composite Membrane for Cardiac Occluders

**DOI:** 10.3390/bioengineering13040422

**Published:** 2026-04-03

**Authors:** Yuqi Li, Yafeng Zou, Xinyi Yang, Wenhai Weng, Lizhen Wang, Yubo Fan

**Affiliations:** 1 Hangzhou International Innovation Institute, Beihang University, Hangzhou 311115, China; by2110116@buaa.edu.cn (Y.L.); lizhenwang@buaa.edu.cn (L.W.); yubofan@buaa.edu.cn (Y.F.); 2 Key Laboratory of Biomechanics and Mechanobiology of Ministry of Education, Beijing Advanced Innovation Center for Biomedical Engineering, School of Biological Science and Medical Engineering, Beihang University, Beijing 100191, China; zzouyafeng@163.com (Y.Z.); 18810938119@163.com (X.Y.)

**Keywords:** ventricular septal defect, polymer-based occluder, shape-memory polymer

## Abstract

Ventricular septal defect (VSD) occluders commonly rely on permanent nitinol frameworks, which may contribute to long-term mechanical mismatch and late complications. Here, we developed a tissue-compliant composite membrane by embedding a 3D-printed poly(vinyl alcohol) (PVA) grid within a shape-memory poly(glycerol dodecanedioate) (PGD) matrix. Grid spacing was varied from 0.1 to 0.5 mm to tune reinforcement density. FTIR indicated that PVA was incorporated mainly through physical interlocking rather than new covalent bonding. The composite preserved near-body-temperature shape recovery. In water at 37 °C, PVA reinforcement increased tensile modulus and fracture strength, although swelling also increased. Finite-element analysis and benchtop occlusion testing consistently showed lower deformation, less strain localization, and smaller bulge height for PGD–PVA than for PGD alone. In vitro assays showed low cytotoxicity, low hemolysis, and prolonged plasma recalcification time. A 12-week pilot degradation study showed that the faster mass loss observed in initial samples was mainly caused by exposed PVA cut edges; after switching to a fully encapsulated design, static mass loss became similar across groups, and dynamic PBS agitation produced about 10% mass loss at 12 weeks. These results support PGD–PVA as a reinforced membrane strategy for polymeric occluders.

## 1. Introduction

Ventricular septal defect (VSD) is among the most common congenital heart diseases and remains a major cause of morbidity in infants and children [1,2]. Large defects that do not close spontaneously can increase left-to-right shunting, elevate pulmonary blood flow and pressure, and, in severe cases, progress to irreversible pulmonary hypertension and heart failure [2]. For defects that require intervention, device closure has become a key option because it can avoid open surgery and shorten recovery time.

Despite broad clinical adoption, VSD occluders must simultaneously achieve secure anchoring, reliable sealing, and long-term mechanical compatibility with the beating myocardium. Computational and experimental studies have shown that occluder performance is highly sensitive to membrane stiffness, thickness, and support architecture, and that excessive deformation or localized strain can compromise sealing stability and safety [3]. Large clinical series have demonstrated the effectiveness of transcatheter closure for muscular and perimembranous VSDs, but device–tissue interaction remains a critical design constraint [4,5]. In particular, late complications such as conduction disturbance and complete atrioventricular block have been reported after transcatheter closure, motivating efforts to reduce chronic irritation and improve compliance [6,7,8].

Most commercial VSD occluders still rely on permanent nitinol wire meshes to provide radial support and shape stability. That design works well clinically, but the metal frame remains in the heart long after endothelialization and may contribute to chronic mechanical mismatch and late complications. To address this issue, partially or fully degradable occluders have been explored using polymeric frameworks and membranes, with encouraging preclinical and early clinical results [9,10,11,12]. Even so, many polymer-based designs still face a practical mechanical problem: they are either too compliant to resist pressure-driven bulging or require a separate stiff support that partly defeats the goal of reducing mismatch.

Biodegradable shape-memory polymers offer an attractive route to reconcile compliance with deployability. Poly(glycerol dodecanedioate) (PGD) is a soft, biodegradable polyester elastomer whose mechanical behavior is closer to that of soft tissue than metal-based devices, and its properties can be tuned through synthesis and curing conditions [13,14,15,16,17]. As a thermally activated shape-memory material, PGD can be programmed into a compact temporary shape at lower temperature and recover near physiological temperature, enabling catheter-based delivery and in situ deployment [18,19,20]. However, PGD alone can be overly compliant and prone to pressure-driven bulging, indicating that internal reinforcement is needed to improve structural stability without sacrificing tissue compliance.

Poly(vinyl alcohol) (PVA) is widely used in biomedical applications because of its favorable biocompatibility, processability, and established use in hydrogel and scaffold systems [21,22,23]. Here we combine PGD with a 3D-printed PVA double-layer grid as an internal support, aiming to create an integrated composite membrane that preserves near-body-temperature shape recovery while improving bulge resistance and reducing strain localization.

In this study, we fabricated co-cured PGD–PVA composite membranes with tunable grid spacing (0.1–0.5 mm). We characterized their chemical structure, thermal transition, and temperature-triggered shape-memory behavior, and measured tensile properties and swelling in water at 37 ± 1 °C. We then used finite-element analysis and an in vitro occlusion test to compare deformation and bulge resistance, followed by cytocompatibility and hemocompatibility assays. The aim was not to present a finished occluder, but to test whether this material combination could strengthen a compliant polymer membrane while retaining near-body-temperature recovery.

## 2. Materials and Methods

### 2.1. Preparation of PGD–PVA Composite Membranes

PGD prepolymer synthesis: dodecanedioic acid and glycerol were mixed at a 1:1 molar ratio in a three-neck flask. The reaction was carried out at 120 °C under nitrogen and then continued under vacuum (−0.08 MPa). Total reaction time was 24 h to obtain a PGD prepolymer.

3D printing of PVA grids: PVA filament (ePVA+, eSUN) was printed using a desktop 3D printer (Adventurer 4, Zhejiang Flashforge 3D Technology Co., Ltd., Hangzhou, China). The printing step was performed in a standard laboratory environment for research-scale material fabrication rather than in a certified medical-device cleanroom. Settings were: nozzle 200 °C, bed 55 °C, nozzle diameter 0.4 mm, and layer height 0.2 mm. A double-layer grid (40 mm × 40 mm × 0.4 mm) was printed. Grid spacing was set to 0.1–0.5 mm in both directions.

Composite formation: PGD prepolymer was melted at 120 °C and poured into a PTFE dish. The printed grid was placed into the melt and vacuum was applied in a vacuum oven (DP310C, Yamato Scientific Co., Ltd., Tokyo, Japan) to improve infiltration. Samples were cured at 120 °C for 3 days to obtain PGD–PVA composite membranes. Final membrane thickness was 0.5, 1.0, or 1.5 mm. For the composite design used in the present study, the printed PVA grid was laser-cut to the target geometry before embedding, and the PGD matrix was prepared with a slightly larger lateral size so that the cut PVA edge remained fully encapsulated after curing.

Naming: composites are referred to as PGD–PVA. Samples are named PVA-0.1 to PVA-0.5, where the number indicates grid spacing (mm). Unless stated, “PVA-x” refers to the corresponding PGD–PVA composite.

### 2.2. Fourier Transform Infrared Spectroscopy (FTIR)

FTIR (7600, Lambda Scientific Systems, Inc., Miami, FL, USA) was used to characterize PGD and PGD–PVA. Samples were ground with KBr and pressed into pellets. Spectra were collected from 4000 to 400 cm^−1^. FTIR was used as a qualitative structural characterization; because no strict replicate number was predefined for this assay, representative spectra are shown for each group.

### 2.3. Differential Scanning Calorimetry (DSC)

DSC (DSC 8000, PerkinElmer, Inc., Waltham, MA, USA)) was used to measure glass transition temperature (Tg). Samples were cooled from 25 °C to 0 °C at 10 °C/min, held for 3 min, then heated to 60 °C at 10 °C/min. Tg was taken from the heating scan (*n* = 5).

### 2.4. Temperature-Triggered Shape-Memory Test

Rectangular strips (30 mm × 15 mm) were cut from PGD and PGD–PVA. Samples were heated to 37 °C and bent in a U-shaped mold, then cooled to room temperature and removed. The fixed angle was recorded as θf. Samples were reheated to 37 °C until stable and the recovered angle was recorded as θr. Angles were measured using ImageJ (version 1.53c, National Institutes of Health, Bethesda, MD, USA) (three repeats per group).

Shape fixity (Rf) and shape recovery (Rr) were calculated as: Rf = (θf/θi) × 100% and Rr = [(θf − θr)/(θf − θ0)] × 100%, where θi is the mold angle during programming and θ0 is the original angle before programming (flat state).

### 2.5. Tensile Testing

Dog-bone specimens were cut by laser (VLS2.30, Universal Laser Systems, Inc., Scottsdale, AZ, USA) from PGD and PGD–PVA (overall length 115 mm; gauge width 6 mm; thickness matched each sample). Uniaxial tensile tests were performed in water at 37 ± 1 °C using a mechanical test system (IPBF-300S, Tianjin Kaier, Tianjin, China). Specimens were pre-soaked in 37 °C water for 24 h. Crosshead speed was 10 mm/min. Elastic modulus and fracture strength were calculated (*n* = 10).

### 2.6. Swelling Ratio

Samples were soaked in deionized water at 37 °C for 48 h (*n* = 3 per group). Wet mass (mw) was measured after gently removing surface water. Samples were then dried to constant mass (md). Swelling ratio was calculated as (mw − md)/md × 100%.

### 2.7. In Vitro Occlusion Performance Test

An in vitro occlusion setup was built. It included pump-driven loading, temperature control, and pressure monitoring. Phosphate-buffered saline (PBS; Beijing Solarbio Science & Technology Co., Ltd., Beijing, China) was driven to impact the sample from the defect opening, creating one-sided pulsatile pressure at 37 ± 1 °C. The defect opening diameter was 10 mm, and the effective clamping diameter was 20 mm.

Samples were fixed on the device and the system was filled with PBS and degassed. After reaching 37 °C, the pressure difference across the sample was set to 200 mmHg at 8 Hz for 10 min. The pump was then stopped while static pressure of 200 mmHg was maintained. While maintaining 200 mmHg, the setup was cooled to below 20 °C to fix the deformed shape. The sample was then removed and the maximum out-of-plane bulge height was measured. Each group was tested three times. This benchtop protocol was designed as a comparative short-term screen of deformation resistance rather than a long-term durability test.

### 2.8. Finite-Element Analysis (FEA)

FEA was performed in Abaqus 2020 to compare relative deformation and strain distribution between PGD and PGD–PVA. A tissue disk (40 mm diameter, 10 mm thickness) with a central defect (6 mm diameter) was used. The outer tissue boundary was fully constrained to provide a simplified reference boundary condition for comparing membrane designs, rather than to reproduce full physiologic myocardial motion. The PGD–PVA membrane was modeled as a cylinder (10 mm diameter, 1 mm height) covering the defect.

Material models: PGD was modeled with a one-term Ogden hyperelastic model (μ = 0.30 ± 0.12 MPa, α = 2.14 ± 0.50, D1 = 0.1333). In the present work, this parameter set was used as an effective material representation for comparative simulations of PGD deformation at 37 °C. The PVA grid was modeled as isotropic linear elastic (E = 2 × 10^9^ Pa, ν = 0.46, ρ = 1230 kg/m^3^) as a first-order representation of the printed reinforcement. The tissue was modeled as an incompressible Mooney–Rivlin hyperelastic material using parameters reported by Jernigan et al. [24] (c10 = −5.84 × 10^4^ Pa, c01 = 6.34 × 10^4^ Pa, c20 = 1.60 × 10^7^ Pa, c11 = −3.53 × 10^7^ Pa, c02 = 1.97 × 10^7^ Pa).

Loading: uniform pressure was applied with a cosine profile. Pressure increased from 0 to 8 kPa in 2 s, was held for 1 s, and then decreased to 0 in 2 s. After unloading, the model ran for another 2 s to capture rebound. The cycle was repeated for 25 cycles. Outputs included maximum principal strain and displacement fields, and axial displacement over cycles.

Note: The FEA geometry and loading were used for qualitative trend comparison rather than one-to-one quantitative prediction. In vitro occlusion testing followed the device geometry and loading described in Section 2.7, so FEA and experiment were compared at the level of deformation trend rather than direct numerical equivalence.

### 2.9. Hemolysis Test

PGD and PGD–PVA (1 mm thick) were rinsed three times with ethanol and three times with PBS, then dried. Fresh anticoagulated rabbit blood (anticoagulant: blood = 1:9, *v*/*v*) was diluted with 0.9% saline at 4:5 (*v*/*v*). Samples were incubated in saline at 3 cm^2^/mL. Saline and distilled water served as negative and positive controls. After pre-incubation at 37 °C for 30 min, diluted blood (0.2 mL) was added to 10 mL saline and incubated at 37 °C for 1 h. After centrifugation in a high-speed centrifuge (ST8, Thermo Fisher Scientific, Waltham, MA, USA) at 3000 rpm for 5 min, the supernatant absorbance at 545 nm was measured using a microplate reader (Multiskan FC, Thermo Fisher Scientific, Waltham, MA, USA). Hemolysis was calculated as [(optical density (OD)sample − ODnegative)/(ODpositive − ODnegative)] × 100%. Each group was tested three times.

### 2.10. Plasma Recalcification Time

Platelet-poor plasma (PPP) was collected by sequential centrifugation of diluted anticoagulated rabbit blood using a high-speed centrifuge (ST8, Thermo Fisher Scientific, Waltham, MA, USA) (5 °C, 1000 rpm, 5 min; then 5 °C, 3000 rpm, 10 min). Washed and dried samples were ground into particles and added to 0.025 mol/L CaCl_2_ at 20% (*w*/*v*). CaCl_2_ in a siliconized tube served as a negative control, and CaCl_2_ in a regular glass tube served as a positive control. After incubating 100 μL CaCl_2_ solution at 37 °C for 30 min, 100 μL PPP was added. Starting 100 s after mixing, tubes were gently tilted every 2 s until clotting was observed. Clotting time was recorded (*n* = 3) and reported as a time ratio versus control.

### 2.11. Cytocompatibility

Sterilization: PGD and PGD–PVA (1 mm thick) were rinsed with ethanol (three times) and water (three times), dried, and exposed to UV light for 60 min. Extracts were prepared by soaking samples in human umbilical vein endothelial cell (HUVEC; Procell Life Science & Technology Co., Ltd., Wuhan, China) medium at 37 °C for 1, 3, or 5 days at 3 cm^2^/mL.

CCK-8 assay: HUVECs were seeded in 96-well plates at 3000 cells/well and cultured for 24 h. Medium was replaced with 100 μL extract (test) or fresh medium (control) (*n* = 5). Cells were cultured with extracts for 1 or 3 days. Then, 10 μL CCK-8 reagent was added and incubated for 1 h in the dark. Absorbance at 450 nm was measured using a microplate reader (Multiskan FC, Thermo Fisher Scientific).

Live/dead staining: HUVECs were seeded on sample surfaces at 1 × 10^6^ cells/sample and cultured for 48 h. Acridine orange/propidium iodide (AO/PI) staining was performed following the kit instructions. Images were acquired with the same channels and exposure settings for all groups.

### 2.12. Statistics

Data are presented as mean ± standard deviation unless otherwise noted. Here, n denotes the number of independently tested specimens or biological replicates for each quantitative assay; for qualitative characterizations such as FTIR, representative data are shown instead of reporting a formal replicate number. Normality was tested with Shapiro–Wilk. One-way ANOVA with post hoc comparisons was used for normal data with equal variance; otherwise, the Kruskal–Wallis test was used. *p* < 0.05 was considered significant.

### 2.13. Pilot Degradation Study

A 12-week pilot degradation study was performed to determine whether the PVA scaffold changed mass-loss behavior and, more specifically, whether edge exposure was responsible for the faster degradation seen in the initial specimens. Two fabrication routes were compared. In the initial route, PGD–PVA composites were cured first and then laser-cut to final geometry, which left PVA cross-sections exposed at the specimen edge. In the revised route, the PVA grid was laser-cut to the target geometry before embedding, and the PGD matrix was prepared with a slightly larger lateral size so that the cut PVA edge was fully encapsulated after curing.

For degradation testing, PGD, PVA-0.3, PVA-0.4, and PVA-0.5 samples were cut with a CO_2_ laser into rectangular blocks (5 × 4 × 1 mm), weighed to obtain the initial mass, and immersed in PBS (*n* = 5 per group). Samples were maintained at 37 °C either under static immersion or on a shaker at 200 rpm. At 2, 4, 6, 8, 10, and 12 weeks, specimens were removed, rinsed with deionized water to remove residual deposits and medium, dried in a fume hood for at least 48 h, and reweighed. Mass loss was calculated as [(m0 − mt)/m0] × 100%. The shaking condition was used as a harsher aqueous screening condition, not as a direct physiological pressure-equivalence test.

## 3. Results

### 3.1. Morphology and FTIR

**Figure 1:**
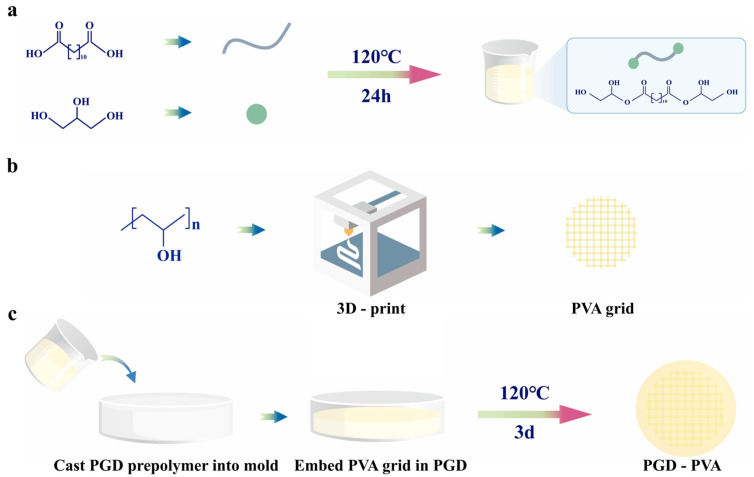
Fabrication workflow for PGD and PGD–PVA, including PGD polycondensation and composite curing. (**a**) Synthesis of the PGD precursor by polycondensation of dodecanedioic acid and glycerol. (**b**) 3D printing of the PVA grid scaffold. (**c**) Composite formation by embedding the printed PVA grid into the PGD precursor, followed by curing to obtain the final PGD–PVA composite membrane.

**Figure 2:**
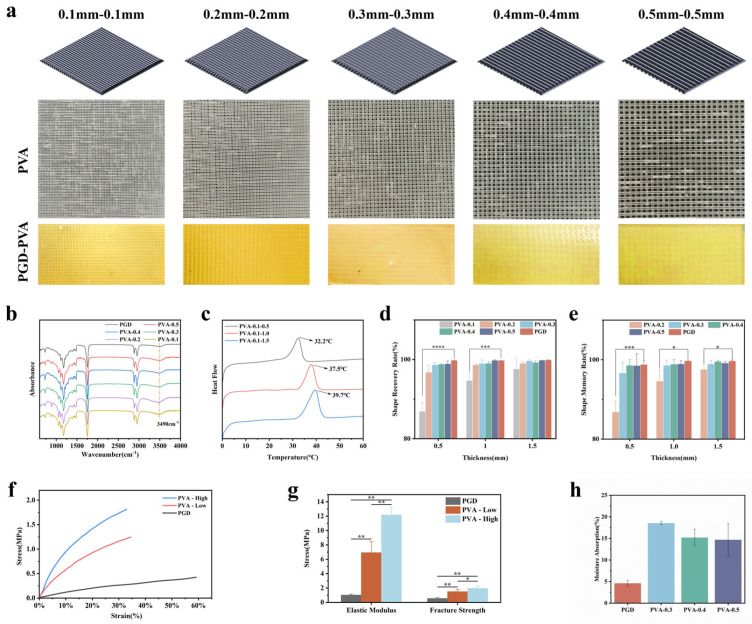
Structure and basic properties. (**a**) Macroscopic images of whole printed PVA grids and the corresponding PGD–PVA composite membranes with different grid spacings. (**b**) Representative FTIR spectra. (**c**) Representative DSC heating curves; Tg values were taken from the heating scan. (**d**) Shape fixity (mean ± SD, *n* = 3). (**e**) Shape recovery (mean ± SD, *n* = 3). (**f**) Stress–strain curves in water at 37 °C. (**g**) Elastic modulus and fracture strength (mean ± SD, *n* = 10). (**h**) Swelling ratio after 48 h at 37 °C (mean ± SD, *n* = 3). **** *p* < 0.0001, *** *p* < 0.001, ** *p* < 0.001, * *p* < 0.05.

Figure 1 summarizes the fabrication of PGD–PVA membranes, including PGD prepolymer synthesis (Figure 1a), 3D-printed PVA grid preparation (Figure 1b), and co-curing after grid embedding (Figure 1c). Macroscopic photographs of the whole printed double-layer PVA grids with spacing from 0.1 to 0.5 mm and the corresponding PGD–PVA composite membranes are shown in Figure 2a. FTIR spectra are shown in Figure 2b. In PGD, the hydroxyl peak near 3331 cm^−1^ decreased and a carbonyl peak appeared at 1735 cm^−1^, consistent with ester formation. In PGD–PVA, a hydroxyl feature near 3490 cm^−1^ remained and increased with higher PVA density. This supports that PVA was incorporated mainly by physical interlocking rather than new covalent bonding.

### 3.2. Glass Transition Temperature

DSC results are shown in Figure 2c. Tg increased with thickness: 32.2 °C (0.5 mm), 37.5 °C (1.0 mm), and 39.7 °C (1.5 mm). The transition is close to body temperature, which supports temperature-triggered deployment (See Figure 2a,b).

### 3.3. Shape-Memory Behavior

Shape fixity and shape recovery are summarized in Figure 2d,e. Recovery decreased slightly as the grid became denser. When grid spacing was ≥0.3 mm, recovery stayed above 98.5% across thicknesses. Even at 1.0–1.5 mm thickness, recovery remained above 95%.

### 3.4. Tensile Properties in Water at 37 °C

Representative stress–strain curves are shown in Figure 2f. Elastic modulus and fracture strength are summarized in Figure 2g, and the corresponding mean ± SD values (*n* = 10) are listed in Table 1. To match the figure labels, two representative designs are reported as PVA-High and PVA-Low (based on the number of PVA fibers aligned with the tensile direction). Elastic modulus increased from 1.03 ± 0.13 MPa for PGD to 6.93 ± 1.56 MPa for PVA-Low and 12.17 ± 1.09 MPa for PVA-High. Fracture strength increased from 0.57 ± 0.10 MPa for PGD to 1.51 ± 0.33 MPa for PVA-Low and 1.95 ± 0.33 MPa for PVA-High, indicating that the reinforcing effect was substantially larger than the specimen-to-specimen variability shown by the error bars.

### 3.5. Swelling

Swelling ratios after 48 h at 37 °C (*n* = 3) are shown in Figure 2h. The presence of the PVA scaffold increased the swelling ratio compared with pure PGD. The PVA-0.3 group reached the highest swelling ratio (about 18%), while PGD was about 4.6%.

### 3.6. Finite-Element Analysis and In Vitro Occlusion Performance

**Figure 3:**
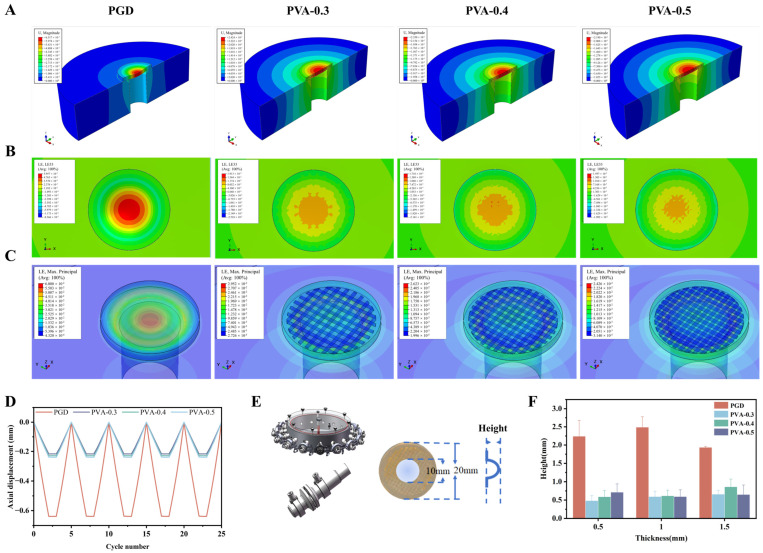
Mechanical stability under defect loading. (**A**–**C**) FEA results showing displacement and maximum principal strain fields for PGD and PGD–PVA under the comparative model conditions. (**D**) Cyclic axial displacement. (**E**) In vitro occlusion setup and maximum out-of-plane bulge height definition. (**F**) Maximum out-of-plane bulge height under static pressure for different thicknesses.

FEA results are shown in Figure 3A–C. Under the same pressure loading, PGD showed larger deformation and stronger localization of maximum principal strain around the defect. PGD–PVA showed lower deformation and less strain localization. Cyclic axial displacement is shown in Figure 3D. PGD had a larger displacement amplitude, while PGD–PVA (PVA-0.3/0.4/0.5) showed smaller and more stable displacement. These simulation results were qualitatively consistent with the lower bulging observed in the benchtop occlusion test.

The in vitro occlusion setup and definition of the maximum out-of-plane bulge height are shown in Figure 3E. The maximum out-of-plane bulge height, fixed by cooling under 200 mmHg static pressure, is summarized in Figure 3F. PGD–PVA showed a much smaller bulge height than PGD. Across thicknesses, the bulge height of PGD was about 2–3 times that of PGD–PVA.

### 3.7. Cytocompatibility of PGD and PGD–PVA Extracts

**Figure 4:**
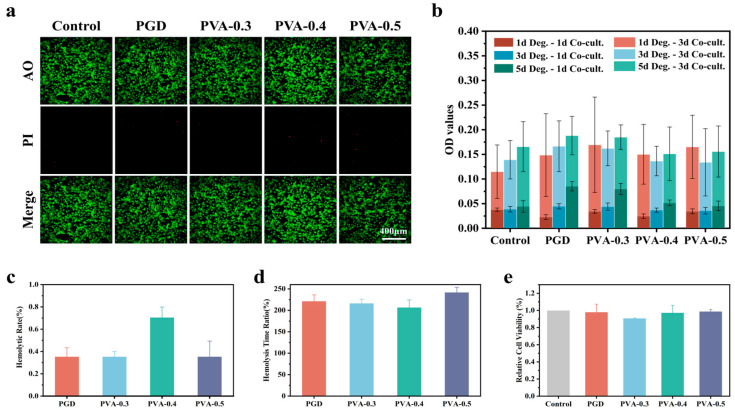
Biocompatibility tests. (**a**) Live/dead staining (acridine orange/propidium iodide, AO/PI) after 48 h. (**b**) CCK-8 results for extracts with different extract and culture times. (**c**) Hemolysis. (**d**) Plasma recalcification time ratio. (**e**) Relative cell viability.

Live/dead staining after 48 h is shown in Figure 4a. Most cells were viable in all groups. CCK-8 results using extracts are shown in Figure 4b, and relative viability is shown in Figure 4e. Extracts prepared after 1, 3, or 5 days did not reduce cell viability compared with the control.

### 3.8. Blood Compatibility

Hemolysis is shown in Figure 4c. Hemolysis was below 1% for all groups. Plasma recalcification time ratios are shown in Figure 4d. PVA-0.3, PVA-0.4, and PVA-0.5 showed longer recalcification times than the control, suggesting weaker activation of coagulation on the material surface under the test conditions (See Figure 4e).

### 3.9. Pilot Degradation Behavior

**Figure 5:**
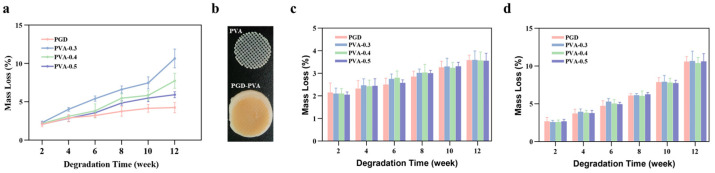
Pilot degradation behavior of PGD and PGD–PVA membranes over 12 weeks. (**a**) Static immersion for the initial fabrication route, in which laser cutting after composite curing left exposed PVA cross-sections at the cut edge. (**b**) Representative photographs of the revised fabrication route, showing the pre-cut PVA scaffold prior to embedding and the corresponding PGD–PVA composite after full encapsulation by a slightly larger PGD matrix. (**c**) Static immersion in PBS at 37 °C for the revised fully encapsulated route. (**d**) Dynamic-agitation degradation in PBS at 37 °C and 200 rpm for the fully encapsulated design.

Figure 5 summarizes the pilot degradation results. In the initial fabrication route, PGD–PVA composites were laser-cut after curing, leaving exposed PVA cross-sections at the specimen edge; under this condition, static mass loss increased with PVA support density (Figure 5a). PGD alone showed the lowest mass loss, whereas the denser PVA-containing groups lost more mass over 12 weeks. Figure 5b shows representative specimens from the revised route, in which the PVA scaffold was cut first and then completely covered by the PGD matrix, thereby eliminating exposed PVA cut edges in the final composite.

Once this fabrication sequence was changed, the static degradation curves of PGD and PGD–PVA became very similar, with no clear group-to-group difference over 12 weeks in PBS at 37 °C (Figure 5c). Under dynamic agitation at 200 rpm, mass loss increased in all groups and reached about 10% by 12 weeks, but the fully encapsulated groups still tracked closely (Figure 5d). Overall, these results indicate that the initially faster degradation was primarily an exposed-edge effect rather than an inherent consequence of embedding a fully encapsulated PVA grid.

## 4. Discussion

Ventricular Septal Defect (VSD) is a prevalent congenital heart anomaly that can severely impair physiological development and quality of life in infants [1,2]. Transcatheter closure using minimally invasive occluders is now widely practiced, with strong clinical evidence for both muscular and perimembranous defects [4,5]. Current commercial devices generally depend on permanent nitinol frameworks to maintain radial support and device geometry, but excessive stiffness and chronic device–tissue contact can contribute to conduction disturbance and late complications such as complete atrioventricular block [5,6,7,8]. These concerns have accelerated interest in degradable or polymer-dominant occluders that reduce permanent foreign material while maintaining adequate acute support [9,10,11,12]. Within that context, the present study focuses on the membrane material itself rather than a complete clinical device system, and therefore does not yet address anchoring, delivery-system compatibility, retrieval capability, or patient-specific interaction with septal anatomy.

FTIR did not show evidence of new covalent bonding between PGD and PVA. We therefore interpret the PGD–PVA interface mainly as physical interlocking of the printed grid within the cured PGD matrix, together with interfacial contact/friction. That appears sufficient for the short-term composite behavior reported here, but it does not resolve long-term resistance to interfacial damage under cyclic loading. The increase in Tg with membrane thickness is most plausibly related to thickness-dependent curing and network development in the PGD phase; local restriction of chain mobility by the embedded PVA may also contribute. Because DSC was performed on cured membranes of different thicknesses rather than on samples with systematically varied chemistry, this interpretation should be read as qualitative. Still, locating Tg near body temperature is advantageous for programming and recovery during catheter-based handling [18,19,20].

Shape recovery remained dominated by the PGD phase. Once the temperature exceeded Tg, PGD provided the recovery force, whereas the PVA grid acted mainly as a passive reinforcement. This likely explains why changing grid density had only a modest effect on recovery under the present conditions, provided that PGD fully encapsulated the scaffold.

Under tensile loading in water at 37 °C, the PVA grid clearly reinforced the membrane, raising both modulus and fracture strength. The quantitative results are now reported directly as mean ± SD from ten specimens per group in Table 1 and Figure 2g. This is relevant because a membrane occluder must remain compliant while still resisting pressure-driven bulging [3,25,26,27,28,29,30]. The trade-off is increased water uptake: the highest-swelling group reached ~18%, which could alter membrane thickness, local fit, or contact pressure in vivo if not controlled. In the present short-term occlusion test, however, this increase in swelling did not cancel the reinforcing effect, because PGD–PVA still showed markedly lower bulge height than PGD alone. Whether repeated swelling and cyclic deformation will promote debonding or sealing instability remains to be tested directly.

The finite-element model was used here as a comparative tool rather than a patient-specific predictor. The outer tissue boundary was fully constrained to provide a simple reference condition, and the PVA grid was modeled as a linear elastic support. Together with the absence of fluid–structure interaction and myocardial anisotropy, these assumptions limit the quantitative predictive value of the model. Even so, the simulations captured the same design trend observed experimentally: internal reinforcement reduced displacement and limited deformation localization.

Durability remains a major open question. A cardiac occluder experiences tens of millions of loading cycles each year. In the degradable occluder literature, this issue is usually addressed either by dedicated fatigue testing of the complete device or by longer implantation/follow-up studies focused on fixation, tissue ingrowth, and gradual absorption [9,11,12]. Our study does not yet reach that stage. The 200 mmHg/8 Hz/10 min pulsatile test and the limited-cycle simulation were used as acute comparative screens for bulging and strain localization, not as lifetime qualification tests. We now state this boundary explicitly. A logical next step is accelerated fatigue testing in a 37 °C aqueous loop while tracking bulge height, stiffness, crack initiation, and damage at the PGD–PVA interface. Because FTIR points to physical interlocking rather than covalent coupling, future interface optimization may also require surface treatment of the PVA grid or other means of improving adhesion.

PGD is a hydrolytically degradable polyester elastomer, and prior work suggests mainly surface erosion with load-dependent modulation of mass loss [16,17,31]. The pilot degradation study clarified one point that was unresolved in the original submission: the faster mass loss seen in the first PGD–PVA samples was largely an edge effect. When the composite was cut after curing, PVA cross-sections were exposed and the PVA-rich groups degraded faster under static immersion. In contrast, Figure 5b shows the revised route, in which the PVA scaffold was trimmed to final geometry before composite formation and then fully covered by a slightly larger PGD matrix. After this change, the static degradation curves of PGD and PGD–PVA became very similar over 12 weeks, and under PBS at 37 °C with slow agitation at 200 rpm the fully encapsulated groups still remained close to one another.

Mass loss alone does not prove retention of mechanical support, but the present result is not obviously at odds with how degradable septal occluders are typically designed. Guo et al. reported a fully degradable VSD occluder intended to maintain robust fixation during the first 3 months and to disappear within 12 months [11]. Li et al. reported a PDO-based VSD occluder in which endothelialization outpaced degradation [12]. Against that background, our 12-week result argues against premature disintegration after full encapsulation, while also making clear what is still missing: time-resolved testing that links mass loss to retained stiffness, cyclic durability, and tissue repair.

At the device level, future work will have to address disk geometry, anchoring, delivery through a sheath, and interaction with different septal anatomies. Imaging-based planning and computational modeling tools are already being used in structural heart interventions to guide sizing and placement [32]. In this context, patient-specific anatomical reconstruction combined with computational simulation could enable virtual evaluation of occluder deployment, including deformation, contact behavior, and interaction with surrounding tissues. Such a framework may support the optimization of the present membrane design by linking material properties and structural configuration to device-level performance under realistic anatomical and loading conditions, once the membrane concept is extended to a complete occluder geometry.

Finally, implantable cardiovascular devices require rigorous biological safety. Our hemolysis and plasma recalcification tests, together with HUVEC extract assays, indicate favorable initial hemocompatibility and cytocompatibility of PGD–PVA. The observed hemolysis below 1% falls within the nonhemolytic range commonly used for blood-contacting biomaterials, and the maintained endothelial cell viability is consistent with the baseline biocompatibility expected for polymeric occluder candidates [33,34,35,36,37,38]. Although direct cross-study comparison is limited because reported polymer occluder studies use different assay formats and endpoints, this initial profile is directionally consistent with the favorable in vitro biocompatibility generally reported for other polymer-based or bioresorbable VSD occluder candidates [9,10,11,12]. These assays do not replace long-term in vivo evaluation, but they support the feasibility of PGD–PVA as a candidate membrane platform for next-generation VSD occluders.

## 5. Conclusions

We prepared PGD–PVA composite membranes by embedding a 3D-printed PVA double-layer grid in a PGD matrix. The reinforced membranes retained shape-memory behavior and showed higher tensile modulus and fracture strength in water at 37 °C, at the cost of increased swelling. FEA and the in vitro occlusion test consistently showed lower deformation and smaller bulge height than PGD alone. In vitro hemocompatibility and cytocompatibility were acceptable. The pilot degradation study further showed that the early acceleration seen in the initial samples was mainly caused by exposed PVA cut edges; after switching to a fully encapsulated design, static mass loss became similar across groups and dynamic PBS agitation produced about 10% mass loss at 12 weeks. These findings support PGD–PVA as a membrane-level material strategy for polymeric occluders, while also defining the work that still needs to be done on fatigue, interface durability, and degradation-support matching.

## Figures and Tables

**Table 1:** Tensile properties in water at 37 °C (mean ± SD, *n* = 10).

Property	PGD	PVA-Low	PVA-High
Elastic modulus (MPa)	1.03 ± 0.13	6.93 ± 1.56	12.17 ± 1.09
Fracture strength (MPa)	0.57 ± 0.10	1.51 ± 0.33	1.95 ± 0.33

## Data Availability

Data is contained within the article.
